# Home care workers caring for adults with heart failure need better access to training and technology: A role for implementation science

**DOI:** 10.1017/cts.2020.36

**Published:** 2020-04-06

**Authors:** Madeline R. Sterling, Nicola Dell, Emily Tseng, Fabian Okeke, Jacklyn Cho, Benedetta Piantella, Jonathan N. Tobin

**Affiliations:** 1 Division of General Internal Medicine, Department of Medicine, Weill Cornell Medicine, New York, NY, USA; 2 Cornell Tech, New York, NY, USA; 3 New York University, New York, NY, USA; 4 Clinical Directors Network, Inc. (CDN), New York, NY, USA; 5 The Rockefeller University Center for Clinical and Translational Science, New York, NY, USA

**Keywords:** Home care workers, home healthcare delivery, heart failure, technology, qualitative

## Abstract

Although highly involved in heart failure (HF) patients’ care, home care workers (HCWs) lack HF training and are poorly integrated into the healthcare team. For its potential to address these challenges, we examined the role of technology among HCWs caring for HF patients. We conducted 38 interviews with key stakeholders. Overall, four themes emerged. Participants reported that technology is critical for HF care, but existing systems are outdated and ineffective. HCWs also have limited access to electronic resources. Technology, training, and principles of implementation science can be leveraged to improve HCWs’ experience in caring for HF patients and home healthcare delivery.

## Introduction

Home care workers (HCWs), which include home health and personal care aides, represent one of the fastest growing sectors of the healthcare industry and US economy [1]. Largely employed by home care agencies which receive Medicare and Medicaid funding, HCWs collectively care for 48 million Americans [2]. HCWs are integral to home healthcare delivery, often assisting patients with personal care, activities of daily living, and serving as minute-to-minute observers of patients’ health [3, 4]. As such, they have the opportunity to respond to and triage patients’ symptoms, which can directly influence patient behavior and outcomes.

Data suggest that up to 25% of adults with heart failure (HF) receive home care [5]. In HF, HCWs prepare low salt meals, monitor weight and blood pressure, offer medication reminders, and assist with doctors’ appointments. Despite this level of involvement, the majority of HCWs have not received HF training, which can impede their ability to recognize and triage patients’ symptoms [6]. Compounding this, our prior research suggests that HCWs often have trouble reaching other members of the healthcare team by phone when their patients are sick [6]. As a result, HCWs may dial 9-1-1, which can lead to trips to the hospital [7]. While some trips are clinically necessary, others could be avoided if HCWs were better supported in the field [7].

Since technology may be able to address some of the challenges, we sought to understand the role of technology among HCWs caring for HF patients and to identify opportunities for how technology could improve home healthcare delivery and better leverage the role of HCWs in the care team.

## Materials and Methods

### Study Design

**Fig. 1.**
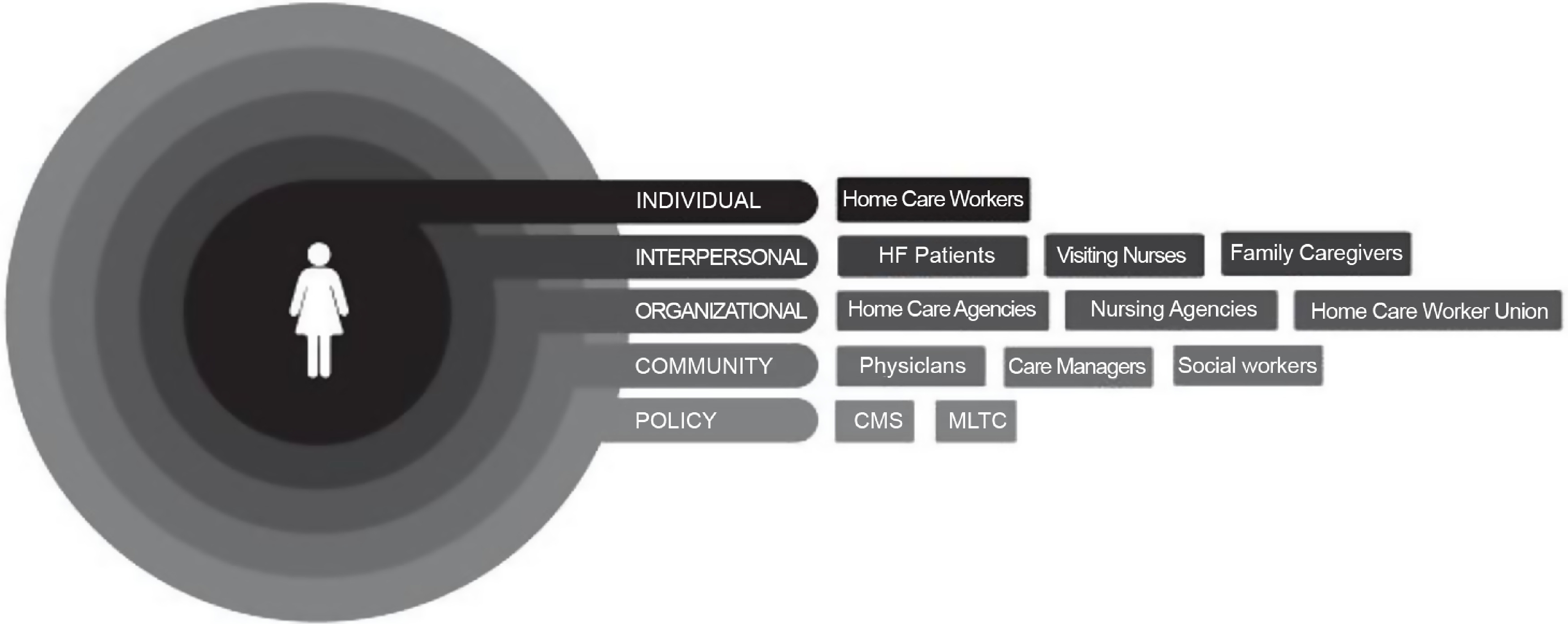
Conceptual model of factors influencing the delivery of care by home care workers to community-dwelling adults with heart failure. Informed by the social ecological model conceptual model of factors influencing the delivery of care by home care workers (HCWs) to adults with heart failure (HF).

This qualitative study was conducted over a 6-month period in 2018 in NYC. We used purposeful sampling and a previously developed conceptual model to recruit key stakeholders who could comment meaningfully on the role of technology among HCWs in HF (Fig. 1) [6]. Overall, eight stakeholder groups were included: HCWs, nurses, home care agency leaders and staff, care coordinators, family (unpaid) caregivers, HF patients, physicians, and social workers. HCWs, nurses, agency leaders, agency staff (clinical and operations), and care coordinators were recruited through a partnership with the Home Care Industry Education Fund (Education Fund), a benefit fund of the 1199SEIU United Healthcare Workers East, the largest healthcare union in the USA. The Education Fund provides training and education to 75,000 HCWs employed by 55 home care agencies across NYC [8]. HF patients, family caregivers, physicians, and social workers were recruited from a large academic medical center. All participants provided verbal or written consent. The study was approved by the Institutional Review Boards at Cornell Tech and Weill Cornell Medicine.

*Figure reproduced with permission from: Sterling MR, Silva, AF, Shaw, A, Leung, P, Jones, CD, Tsui, E, Robbins, L, Lee, A, Escamilla, Y, Wiggins, F, Sadler, F, Shapiro, MF, Charlson, ME, Kern, LM, Safford, MM. “It’s Like They Forget That the Word ‘Health’ is in ‘Home Health Aide:” Understanding the Perspectives of Home Care Workers Who Care for Adults with Heart Failure: Understanding the Perspectives of Home Care Workers Who Care for Adults with Heart Failure. J Am Heart Assoc. 2018; 7: e010134*.

### Data Collection

Three researchers (MRS, BP, JC) conducted interviews using a semi-structured topic guide. Questions focused on the role of technology in the workflow of HCWs caring for HF patients in the home. Interviews were conducted 1:1 or in a focus group, depending on the preference of the stakeholder. Interviews were audio-recorded and professionally transcribed.

### Data Analysis

Two members of the research team (MRS, ND) led the analysis and three members (JC, BP, ET) coded the data. Data were analyzed thematically, an approach used in health-related research when a conceptual model already exists [9]. Since the model did not focus on technology, we took a flexible approach to coding, using established codes when appropriate, but adding ones when new concepts arose [10]. Coding occurred alongside interviews. Three team members (JC, BP, ET) independently reviewed the first transcript. Under the guidance of two members (MRS, ND), a codebook was developed. The codebook was applied to the first transcript and subsequent transcripts by the three coders. The entire team met every fifth transcript to reconcile coding differences. Codes with similar properties were collapsed into categories, which were further consolidated into themes. Data collection ended when no new themes emerged [11]. Themes were discussed and refined with other team members (FO, JNT) who had not conducted or coded interviews, but who had content expertise [12].

## Results

A total of 38 participants were recruited (Table 1). HCWs, agency staff and leaders, and care coordinators worked for nine different home care agencies in NYC. HCWs were all women, ranged in age 22 to 69 years, and had on average 13 years of caregiving experience. Interviews lasted between 30 and 60 min. Overall, four major themes emerged. Each theme is described below, with representative quotations shown in Table 2.

**Table 1.** Study participants

Stakeholder type	N	Interview type
Agency leaders and staff	10	FG (2) and II
Care coordinators	1	II
Family caregivers	2	II
Home care workers	12	FG (2)
Patients	4	II
Doctors	2	II
Visiting nurses	6	FG (1) and II
Social workers	1	II

FG: focus group; II: individual interview(s).

Study participants by stakeholder type; participants represent eight different stakeholder groups.

**Table 2.** Major themes and illustrative quotations

Theme 1: Communication is critical but problematic	“The way it works is that the home care worker observes a change in the patient’s condition. Call a call center. The call center sends it to the coordinator. The coordinator then sends it to the managed long-term care plan contractor who would send it to a nurse and social worker. As you can imagine, all of this takes a long time.” (Nurse)
	“HCWs are not recognized members of the team… they need to be empowered to observe and report what they see.” (Agency Leader)
Theme 2: Existing technology is outdated and ineffective	“It’s a very clunky scheduling and task system.” (Agency Leader)
	“I realized the worker was recording the blood pressure…she would write the numbers (down) but then it’s not in the machine, or transmitted anywhere.” (Home Care Worker)
Theme 3: HCWs have limited access to disease-specific electronic resources	“(We) look up information about HF on Google a lot.” (HCW)
	“I think (a HCW) having supplementary materials for a heart failure patient …is absolutely necessary. (Agency Staff)”
Theme 4: Technology can be leveraged to improve HCWs’ experience and healthcare delivery	“We were part of a pilot with a few other agencies. The pilot created more coordination and support between the HCW and a coordinator and a clinical care manager, who is the nurse. That made a big impact. What was good was that it enabled the clinical problems in the field to be triaged better. So I think the workers felt more supported and their questions could be answered. See, there’s an opportunity to leverage and utilize the home care worker. Incorporating nursing staff is very important piece of this too, I should mention.” (Agency Leader)
	“We need to leverage all the data we collect, as clunky as it is, in some instances non-clinical as it, but really important to the healthcare space.” (Agency Leader)

HCW, home care worker.

Four major themes and representative quotes from study participants.

### Theme 1: Communication Is Critical but Problematic

Participants reported that home health care for HF patients often involves many healthcare professionals and requires effective communication among these professionals about patients’ health and health care. However, this infrequently happens. Rather, information is slowly and imperfectly communicated by telephone among HCWs, nurses, clinicians, and care coordinators, with response times varying from hours to days among these providers, which can delay decision making for patient care in the home. HCWs reported that they rarely felt like true members of the patients’ care team, which made effective communication all the more challenging. For example, not only were they often unable to reach their supervising nurses (at their agencies) by phone in a timely manner but they were also unsure if they should call patients’ doctor(s) during emergencies.

### Theme 2: Existing Technology Is Outdated and Ineffective

Participants reported that many aspects of HCWs’ day-to-day care of HF patients involved digital and nondigital technology. For example, at the beginning of each patient assignment, HCWs review a Care Plan, which is a paper document that resides in the patients’ home. The Care Plan, which is drafted by the visiting nurse and signed by the patients’ doctor, guides care in the home and outlines the tasks for which HCWs are responsible. While some agencies provide these electronically, most use a multipart carbonless paper format. The document, for example, may ask a HCW to take a patient’s blood pressure or weigh them daily. HCWs explained that while they write these values down on paper, the data often stay in the patients’ home (on the Care Plan or another sheet of paper) and may or may not be physically transmitted to other members of the healthcare team or to their agency, which can be problematic if values are abnormal. Participants also explained that agencies require HCWs to “punch in” to work using an interactive voice response telephone system. While “punching out” at the end of the shift, the system also queries them (yes/no) about care tasks completed, which do not always correspond to the Care Plan. Thus, although they are using technology to log attendance and hours, it is not being used to leverage their observations of patients or to transmit symptom-related information to the rest of the care team.

### Theme 3: HCWs Have Limited Access to Disease-Specific Electronic Resources

Although HCWs receive general health training, participants reported that most HCWs have not received training on HF, a condition for which they frequently provide care. This lack of training may limit their HF knowledge and caregiving abilities. Additionally, they have limited access to electronic resources on HF in the home. That is, if a patient is experiencing a new symptom – such as shortness of breath – they do not have information on HF at their fingertips to consult. Many described searching the Internet for HF signs and symptoms by searching Google with their smartphones. This may be problematic, as connectivity in patients’ homes is limited (or absent), and undirected web searches may not generate reliable medical information. Also, HCWs expressed frustration with using their personal mobile devices for work-related matters.

### Theme 4. Technology Can be Leveraged to Improve HCWs’ Experience and Healthcare Delivery

Participants felt that technology could address some of the challenges that HCWs face (e.g. lack of training and communication challenges), while also improving healthcare delivery for HF patients. One idea was for agencies to adopt text-based messaging which would enable HCWs to report their clinical observations and receive feedback in real time. Participants explained that for this to work, though, sufficient buy-in and responsiveness on the receiving end of such texts (via agency nurses and staff) would be needed. This concept would also need to be introduced to patients and family members as well; otherwise, texting by HCWs could be inappropriately perceived as nonwork related. Another idea was to create an online portal in which HCWs could access disease-specific information, such as signs and symptoms of HF. Finally, nearly all participants expressed a desire for technology to help integrate healthcare team members and their corresponding data and tasks.

## Discussion

Despite being integral to home healthcare delivery, we found that HCWs lack HF training and are poorly integrated into the medical team, a phenomenon worsened by ineffective communication practices. We also found that while HCWs often utilize technology to care for patients, the majority of tools and systems they use are outdated, do not meet their current needs, and exacerbate communication challenges. In light of this, stakeholders proposed new ways in which technology could be leveraged to improve the experience and workflow of HCWs, and in turn, improve patient care.

The challenges we identified among HCWs speak to some of the broader inequities that this workforce faces. Although HCWs spend more time with patients than any other medical provider, they are often invisible to other providers [13]. There are many possible reasons for this. First, HCWs, most of whom are women and racial/ethnic minorities and may have been born outside the USA, are composed of structurally disadvantaged populations who struggle to earn fair wages and long-term employment [1, 14, 15]. Second, there is a systematic lack of awareness of their role by other providers. Third, working conditions can be poor and isolating [16, 17]. Despite this, studies have shown that HCWs generally like their jobs, find them meaningful, and can have a positive impact on patient outcomes, when utilized at their skill level [18, 19].

Our findings also demonstrate how the principles of implementation science could be used to leverage technology and advance the role of HCWs in the context of HF care. Implementation science can advance health equity when practices are implemented across the healthcare system, particularly among disadvantaged populations [20, 21]. Thus, a key principle is to ensure that key stakeholders are at the table. Although numerous tech-based interventions in HF exist, none have been designed with the HCWs’ needs in mind or are intended to be delivered by HCWs themselves. Since HCWs are with patients for prolonged periods of time and are astute observers of patient care, a first step would be for existing home-based interventions in HF would be to incorporate HCWs’ recommendations regarding ways to overcome existing communication challenges. Second, the Health Equity Implementation Framework, which has been designed specifically for vulnerable populations [22], could be utilized to design and evaluate tech-based interventions for HCWs, which would ensure that innovations are acceptable and feasible for HCWs and patients and also account for HCWs’ workflow. Finally, it is likely that different strategies will be required to address different challenges. For example, while a text-based messaging application may allow HCWs to more reliably reach their supervisors to report clinical observations, an online HF resource may enable HCWs to access knowledge and subsequently feel more confident caregiving. Thus, comparative effectiveness research to test different implementation strategies head-to-head, or SMART designs that utilize adaptive implementation strategies, may be warranted [23].

### Limitations

We recruited HCWs and home care agencies leaders from unionized agencies, which limits our ability to generalize our findings to privately employed or nonunionized populations. Interviews occurred by both focus groups and one-on-one interviews, which may have introduced selection bias. Additionally, this study was conducted in NYC, so findings may not apply to small cities or rural areas.

## Conclusion

Although integral to home healthcare delivery for adults with HF, we found technology use among HCWs to be suboptimal. In addition to identifying several challenges, we also identified several opportunities for how technology and training could improve HCWs’ ability to care for HF patients in the home. Implementation science has the potential to not only advance the equity of the workforce but also can leverage HCWs as a vehicle for change in patient care and the healthcare system. The lessons learned from better integrating HCWs into the health care team for the management of HF can be extended to other chronic diseases, which will be critical to meet the complex long-term care needs of an aging and clinically complex population.
